# Medial tibial plateau sustaining higher physiological stress than the lateral plateau: based on 3D printing and finite element method

**DOI:** 10.1186/s12938-022-01039-x

**Published:** 2022-09-16

**Authors:** Liqin Zheng, Yuexing Dai, Yongze Zheng, Xingpeng He, Minhui Wu, Desheng Zheng, ChiHung Li, Yueguang Fan, Ziling Lin

**Affiliations:** 1 The First Clinical Medical College, Guangzhou University of Chinese Medicine, Guangzhou, China; 2 Department of Orthopedics, Puning Hospital of Traditional Chinese Medicine, Jieyang, China; 3 International College, Guangzhou University of Chinese Medicine, Guangzhou, China; 4 Department of Joint Surgery, First Affiliated Hospital of Guangzhou University of Chinese Medicine, Guangzhou, China; 5 Department of Orthopedic Trauma, First Affiliated Hospital of Guangzhou University of Chinese Medicine, Guangzhou, China

**Keywords:** Knee, Femoral condyle trabeculae, 3D printing, Finite element method, Mechanical properties

## Abstract

**Background:**

Medial compartment knee osteoarthritis (KOA) accounts for most KOA cases, and increased trabecular bone volume fraction (BV/TV) is one of the pathological changes in the tibial plateau of KOA. How BV/TV changes before and after the menopause and its effects on medial compartment KOA are yet to be clarified.

**Methods:**

Twenty femurs from twenty 12-week-old rats were included. The operated group underwent ovariectomy (to represent the osteoporosis condition), called the O group, and the non-operated group was the normal control, called the N group. Micro-CT scans of the femoral condyles were acquired 12 weeks after the surgery, and the volume of interest (VOI) of medial-, inter-, and lateral-condyle trabeculae were three-dimensional (3D) printed for uniaxial compression mechanical test and simulated by the finite element (FE) method.

**Results:**

The results demonstrated that the O group indicated poorer trabecular architecture than the N group in three parts of the femoral condyle, especially in the intercondyle. Within the group, the BV/TV, trabecular thickness (Tb.Th), and trabecular number (Tb.N) ratios between the medial and lateral condyles were greater than 1 in both N and O groups. The medial condyle trabeculae's mechanical properties were higher than those of the lateral condyle, and this superiority appears to be broadened under osteoporotic conditions. FE modelling well reproduced these mechanical differentiations.

**Conclusions:**

According to Wolff's law, the higher BV/TV and mechanical properties of the medial femoral condyle may be due to inherent imbalanced loading on the knee component. Alterations in BV/TV and their corresponding mechanical properties may accompany KOA.

## Introduction

Trabeculae play a critical role in loading transfer and energy absorption within bones and across joints [1, 2]. The three-dimensional (3D) microarchitecture of trabeculae reflects a balance between the minimal metabolic cost of maintenance and maximal network stability under multidirectional loading conditions [3]. Previous studies have consistently suggested that trabecular bone volume fraction (BV/TV) is the single best determinant of mechanical properties [4–8]. Microarchitecture and bone mass (particularly BV/TV) are two significant aspects that determine trabecular mechanical properties compared to tissue properties [9]. When BV/TV is combined with fabric anisotropy (DA), more than 90% of the trabecular tissue elastic properties variance can be explained [7, 10]. Numerous studies have described the power-law relationships between trabecular modulus and strength in samples pooled from multiple sites spanning a wide range of densities and tissue properties, while it appeared to be linear within a single anatomic site [11]. Fracture and non-fractured proximal femurs exhibit different regression relationships between the modulus and BV/TV and modulus and trabecular number (Tb.N), indicating that the 3D spatial arrangement and attenuation of trabeculae could jeopardise the apparent mechanical properties and whole bone strength [12, 13].

The spatial attenuation of trabeculae is closely related to various bones and joint degenerative processes. Trabeculae have a large surface-to-volume ratio, where a higher bone turnover and remodelling occur after osteoporosis (OP), and the modulus and strength deteriorate by approximately 10% per decade [14, 15]. Evidence has shown that different types of musculoskeletal disorders (for example, osteoarthritis, osteoporosis, and rheumatoid arthritis) demonstrate diverse morphometric characteristics of trabeculae, leading to different mechanical properties [8]. For OP, trabecular tissue decreases in mass primarily by thinning of plate-shaped into rod-shaped trabeculae [8, 16], while osteoarthritis (OA) demonstrates a higher BV/TV and trabecular thickness (Tb.Th), and lower structure model index (SMI). OA also has inferior mechanical properties in the early stage compared to OP [17, 18]. However, as OA progresses, it eventually develops into tissues with poor 3D microarchitecture, both in histomorphology and mechanical properties, and may lead to deformities.

Knee osteoarthritis (KOA) is the most common OA, with varus deformities accounting for most deformity patterns [19, 20]. Various deformities are characterised by a mechanical axis < 180° on full-leg standing anteroposterior radiographs and a narrowed medial compartment. Biomechanical factors such as microarchitecture and loading play a significant role in the progression of various deformities [21–23]. Furthermore, one study found that the medial tibial plateau anatomical level was lower than the lateral plateau in medial compartment KOA, which has been described as a "non-uniform settlement" of the tibial plateau [24, 25]. An asymmetrical distribution of trabeculae in the tibial plateau and proximal fibula support may be an essential anatomic factor leading to "non-uniform settlement" of the tibial plateau [26]. However, most of these changes were observed in KOA patients or related to tibial anatomy. Whether trabeculae alteration (or, specifically, mechanical property alteration) accompanies, precedes, or follows KOA has yet to be clarified. This limited information on KOA is partly due to the interference from the irregular tibial plateau structure (i.e. asymmetry of medial and lateral plateau, fibula support) and the variant arrangement of trabeculae during KOA progression. The knee joint consists of the proximal tibial and distal femoral, and the femoral condyle trabeculae structure indirectly reflects the loading condition on the tibial plateau while insusceptible from anatomic asymmetry; therefore, investigating the loading transfer from the femur perspective is ideal and indispensable.

This study aimed to better elucidate the spatial arrangement and attenuation of trabeculae within and across femoral condyles. The study also highlights the resultant mechanical properties before and after menopause to reveal the physiological loading distribution on the tibial plateau indirectly. Thus far, we performed a series of parameter studies to quantify the microstructure of a rat cohort and developed 3D printing trabeculae and micro-CT-based finite element (FE) models to investigate the mechanical discrepancy. Specifically, our objectives were to (1) confirm the trabecular structural variations and attenuation within the distal femoral condyle dependence of the anatomic site and (2) determine the variations and attenuation of mechanical properties. Accurate 3D printed models of a cohort of trabeculae distinguish this study from previous studies and provide a substantial degree of closure to this clinical issue.

## Results

### Structural parameter differentiation

**Fig. 1:**
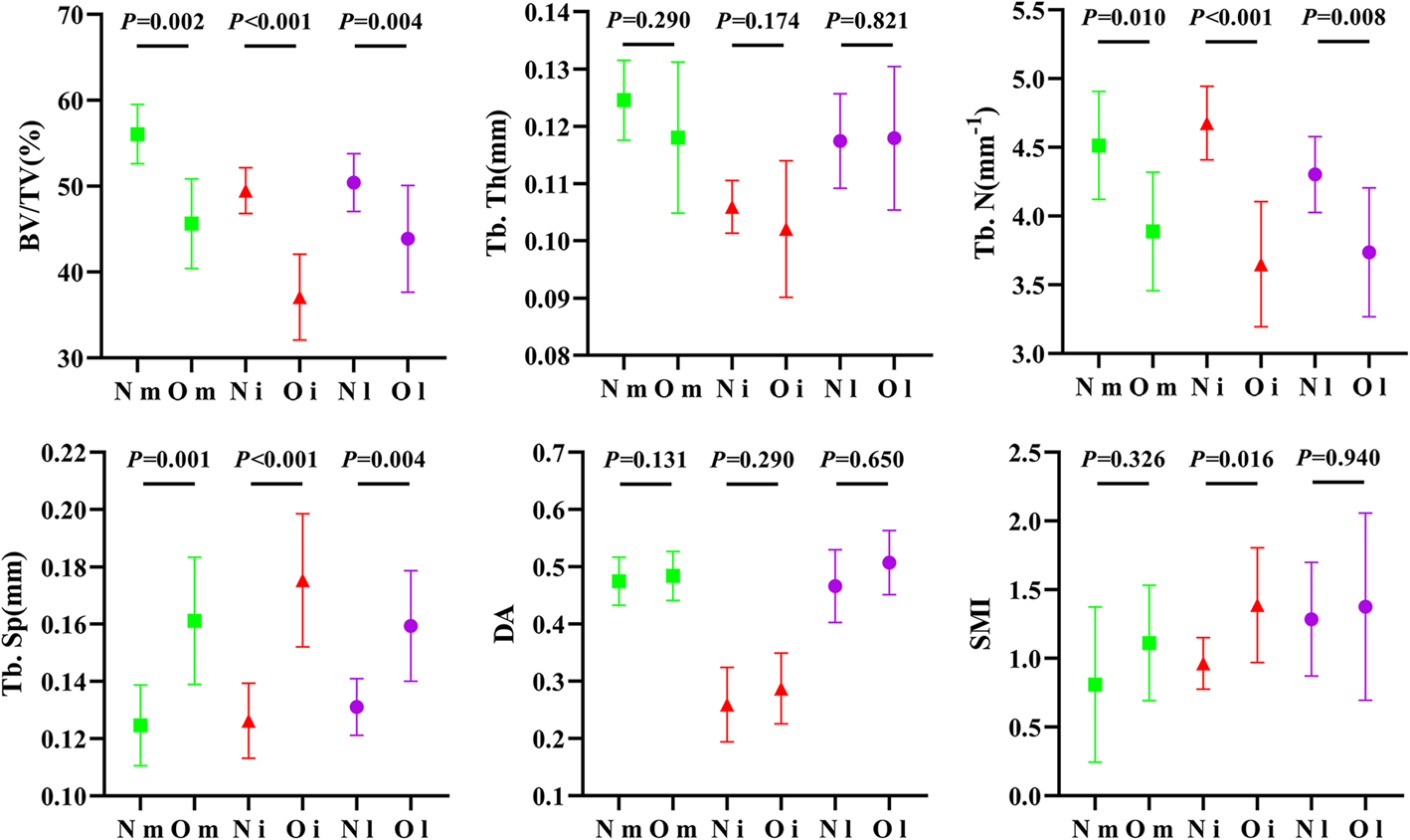
Structural comparison between N and O group in medial-, inter- and lateral-condyle

The O group demonstrated significantly lower BV/TV (P < 0.01), Tb.N (P < 0.05), and higher trabecular separation (Tb.Sp) (P < 0.01) than the N group in the three parts of the femoral condyle, while no significant differences were observed in Tb.Th and DA. There was a significantly higher SMI in the O group only in the intercondyle (P < 0.05) Figure 1.

**Fig. 2:**
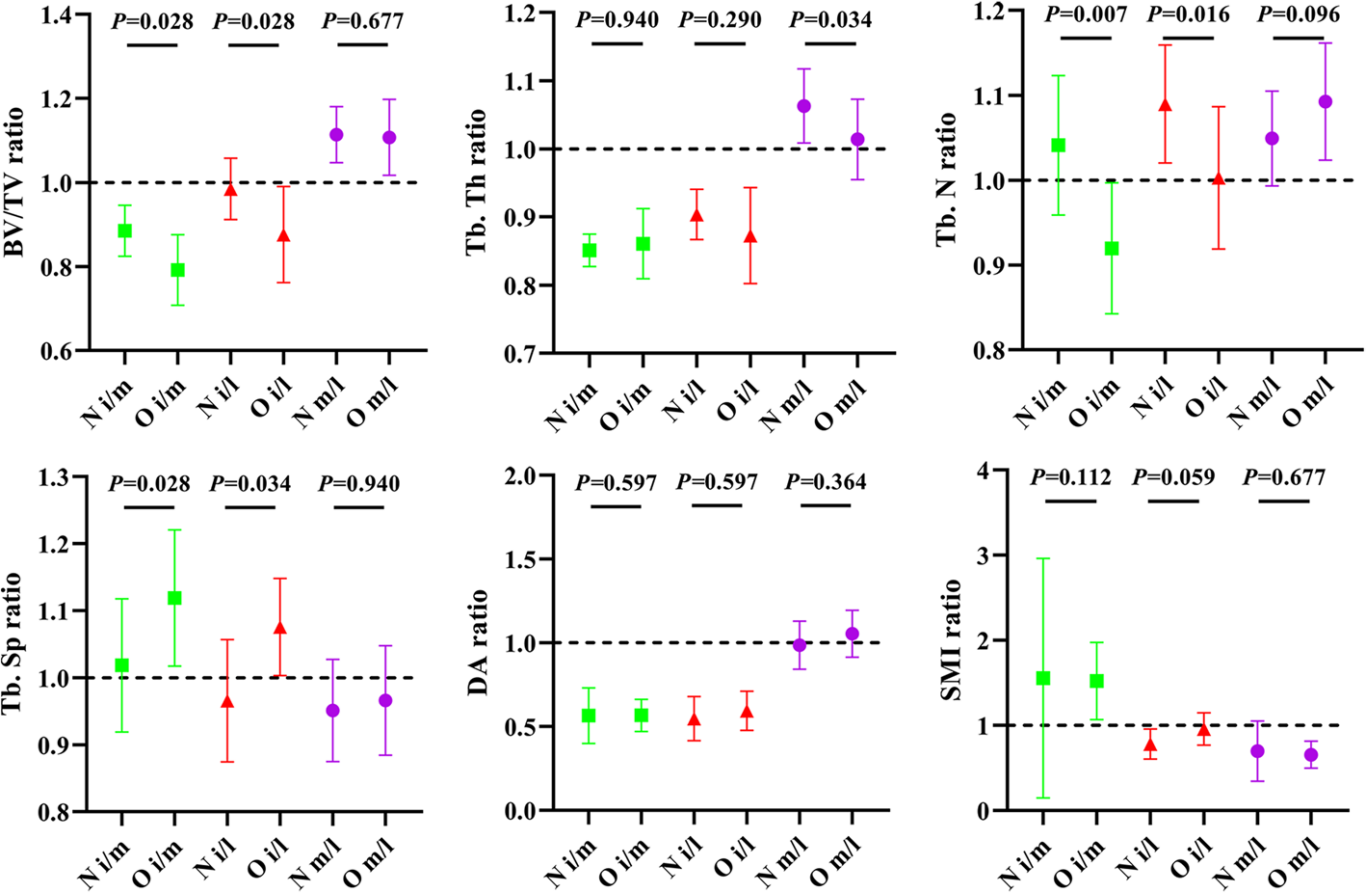
Structural ratio of inter/medial (i/m), inter/lateral (i/l), and medial/lateral (m/l) condyle between the N and O group

We defined the structural ratio of the inter/medial (i/m), inter/lateral (i/l), and medial/lateral (m/l) condyles to facilitate comparison. A ratio of > 1 indicates that the numerator is greater than the denominator. As shown in Fig. 2, the medial femoral condyle demonstrated higher BV/TV, Tb.Th and Tb.N (structural ratio > 1), but lower Tb.Sp and SMI (structural ratio < 1) than the lateral femoral condyle in both the N and O groups. No significant differences were found in the medial/lateral structural ratio (m/l) between the N and O groups, except for the Tb.Th ratio. Compared to the lateral and medial condyles, the intercondyle showed lower BV/TV, Tb.Th, and DA in both normal and ovariectomised conditions (structural ratio < 1) but a lower Tb.N and higher Tb.Sp only after ovariectomy. The SMI ratio appeared to be constant in the three-part anatomy, regardless of ovariectomy. These changes suggest that intercondyle trabeculae suffer from higher bone absorption than the lateral and medial condyles. Almost all structural parameters and parameter ratios demonstrated a higher standard deviation in the O group, indicating large variations among individual subjects in this group.

Collectively, we could observe that BV/TV, Tb.N, Tb.Th, Tb.Sp, and their ratios among the three femoral condyles differed significantly under ovariectomised conditions. In addition, we found that the medial femoral condyle trabeculae appeared to be more abundant than the lateral ones.

### Three-dimensional printing (3DP) trabeculae accuracy validation

As shown in Table 1, in both the N and O groups, the weight of 3DP volume of interest (VOI) trabeculae was found to significantly correlate with their BV/TV (R^2^ > 0.96, P < 0.001), indicating that 3DP VOI trabeculae replicated the actual structure well. The table shows that the 3DP medial condyle trabeculae were heavier than the lateral condyle trabeculae, which corresponded to the finding that higher BV/TV was dominant in the medial condyle.

**Table 1:** Accuracy validation between 3DP VOI trabeculaes and BV/TV

Group	Anatomy	Weight (g) (${\overline{\text{x}}} \pm {\text{s}}$, n = 10)	BV/TV (%) (${\overline{\text{x}}} \pm {\text{s}}$, n = 10)	R^2^	P
N	Medial condyle	4.48 ± 0.35	56.05 ± 3.45	0.965	< 0.001
	Inter-condyle	3.91 ± 0.23	49.49 ± 2.65	0.984	
	Lateral condyle	3.95 ± 0.33	50.42 ± 3.37	0.966	
O	Medial condyle	3.64 ± 0.48	45.64 ± 5.21	0.963	
	Inter-condyle	2.83 ± 0.42	37.08 ± 4.99	0.984	
	Lateral condyle	3.30 ± 0.45	43.88 ± 6.22	0.987	

### Mechanical properties differentiation

**Fig. 3:**
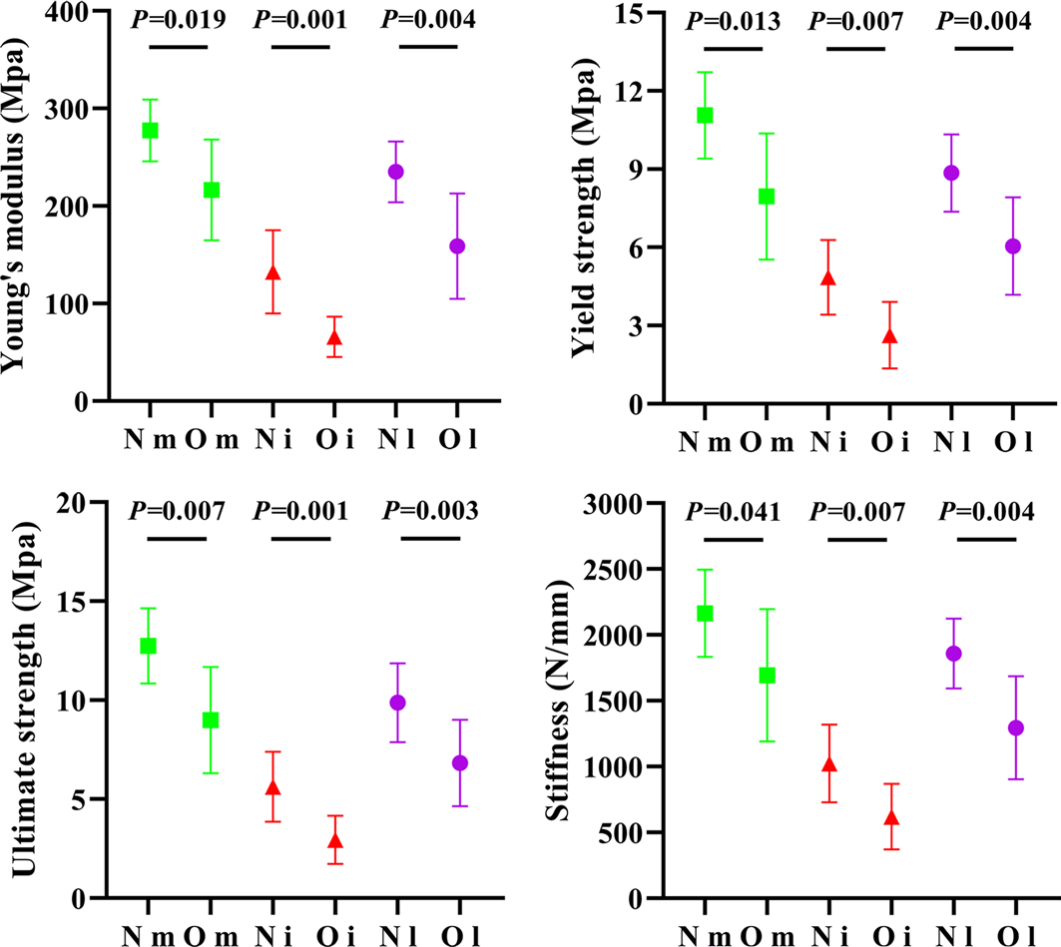
Mechanical properties comparison of 3DP medial-, inter-, and lateral-condyle trabeculae in N and O group

The O group demonstrated a significantly lower Young’s modulus (P < 0.05), yield strength (P < 0.05), ultimate strength (P < 0.05), and stiffness (P < 0.05) than the N group in the three condyles. In both the N and O groups, the average mechanical properties of the medial condyles were the highest, followed by those of the lateral condyles and intercondyles. This differentiation is in line with the aforementioned BV/TV trends, as shown in Fig. 3.

**Fig. 4:**
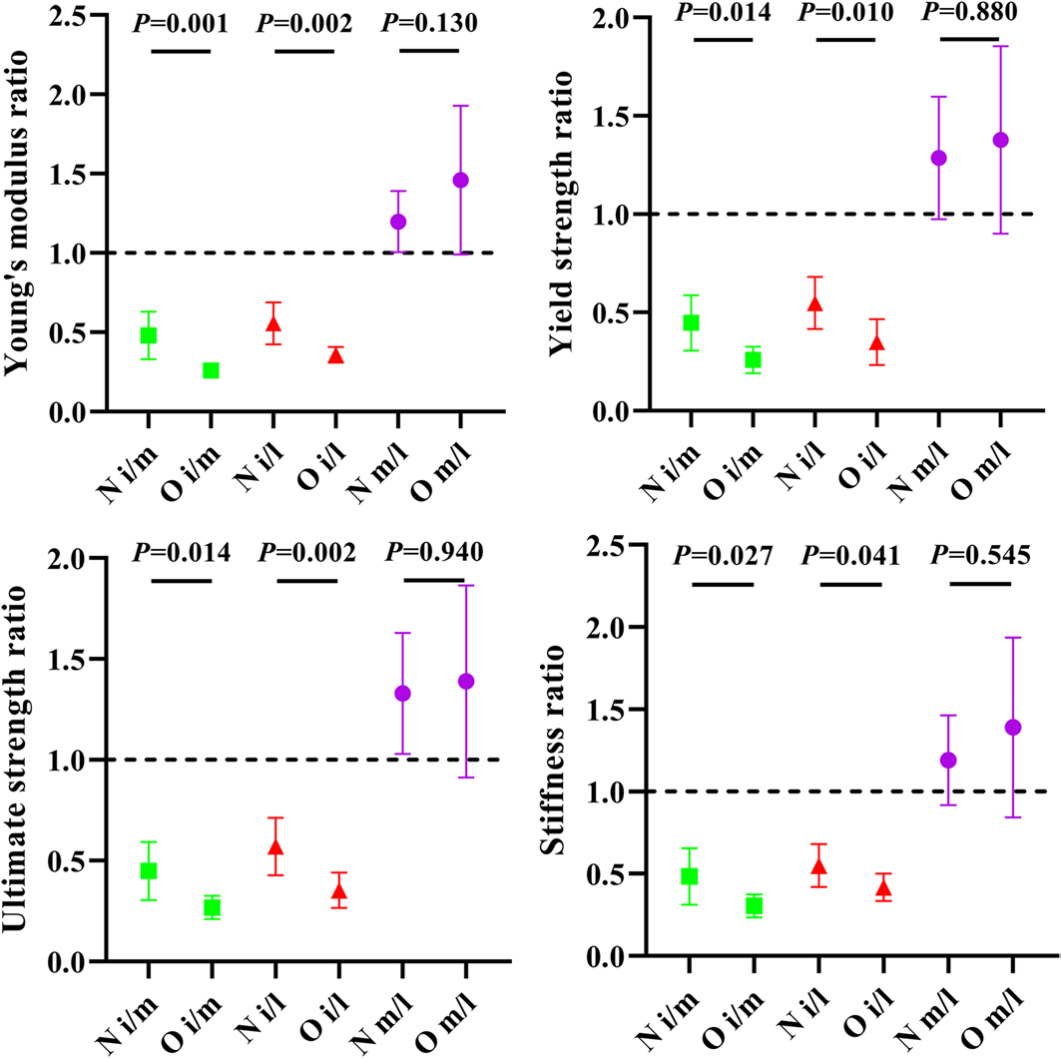
Mechanical properties ratio of inter/medial (i/m), inter/lateral (i/l), and medial/lateral (m/l) condyles between the N and O groups

All mechanical property ratios of medial/lateral (m/l) were higher than 1 regardless of ovariectomy, indicating that medial condyle trabeculae were more mechanically tougher than the lateral condyle trabeculae, whereas the medial/lateral (m/l) ratio within the O group seemed to be slightly higher than that of the N group, but the difference was not significant (P > 0.05). As expected, the intercondyle trabeculae demonstrated the lowest mechanical content among the three anatomies (i/m ratio < 1, i/l ratio < 1). The O group demonstrated a lower mechanical property ratio of inter/medial (i/m) and inter/lateral (i/l) compared to the N group, suggesting that more severe mechanical attenuation occurred in the intercondyle, as shown in Fig. 4.

Comparing the O and N groups, the reduction of BV/TV in the medial condyle (16.07%) was not significantly different from that of the lateral condyle (15.75%); however, the reduction in the mechanical properties of the medial condyle was smaller than that of the lateral condyle, especially in Young's modulus and stiffness, indicating that the asymmetric reduction of mechanical properties occurred between the medial and lateral femoral condyles. The BV/TV and mechanical property reduction of the intercondyle were the highest, as shown in Table 2.

**Table 2:** Reduction of BV/TV and mechanical properties of O group compare to N group

Anatomy	Properties	BV/TV reduction (%)	Properties reduction (%)
Medial condyles	Young’s modulus	16.07	22.02
	Yield strength		28.16
	Ultimate strength		29.40
	Stiffness		21.76
Inter-condyles	Young’s modulus	25.06	50.32
	Yield strength		45.75
	Ultimate strength		47.76
	Stiffness		39.51
Lateral condyles	Young’s modulus	15.75	32.42
	Yield strength		31.71
	Ultimate strength		30.85
	Stiffness		30.37

### FE analysis

**Fig. 5:**
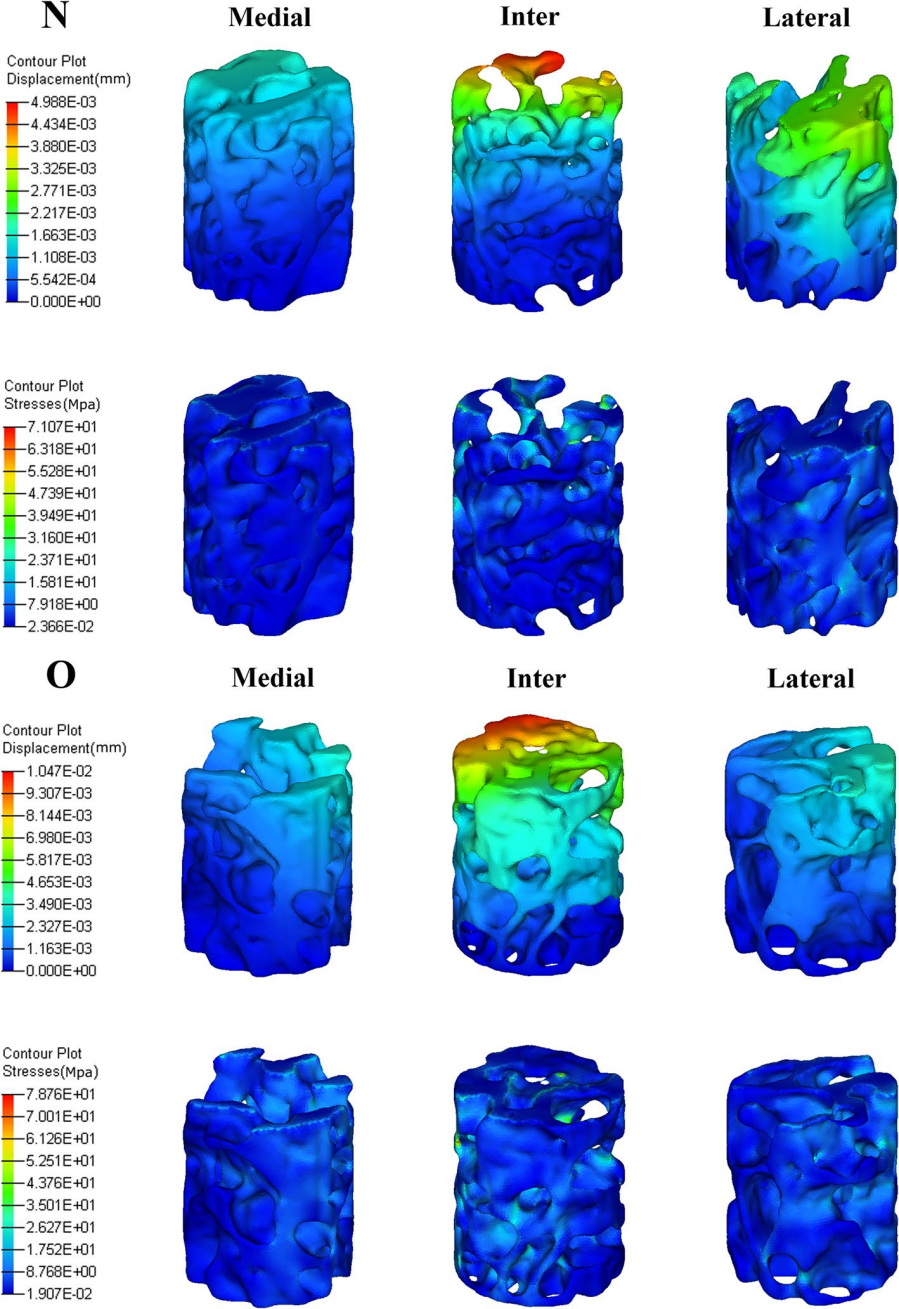
Displacement and stress nephogram of VOI trabeculae in the N and O groups

As shown in Fig. 5, intercondyle trabeculae in both the N and O groups generally demonstrated higher displacement than the lateral condyle trabeculae. The medial condyle trabeculae showed minimum displacement. Similarly, there were more stress concentration regions within the intercondyle trabeculae than within the lateral condyle trabeculae. The medial condyle trabeculae showed a region of minimal stress. It is readily comprehensible that a weaker structure deforms and sustains a higher stress concentration more easily.

**Fig. 6:**
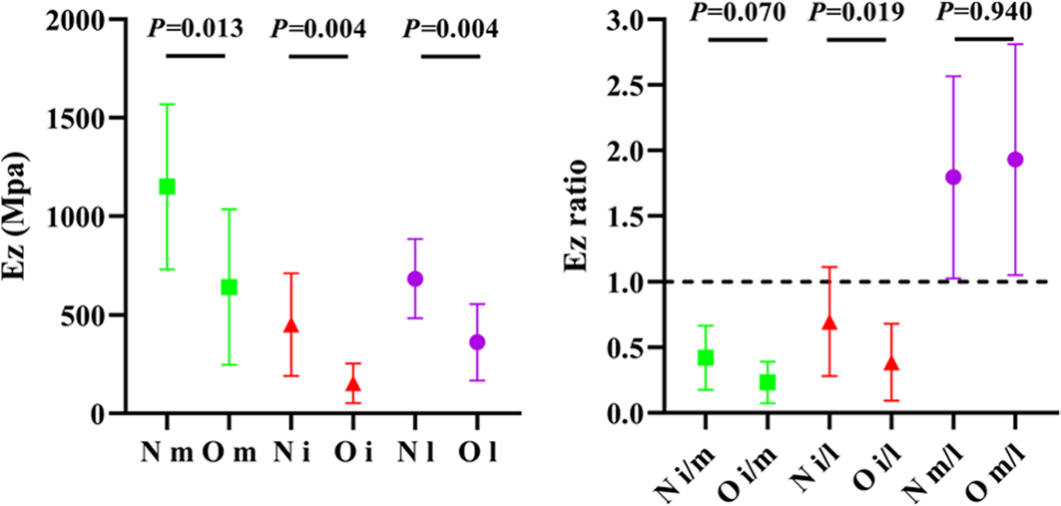
Ez of medial-, inter-, and lateral-condyles and their ratio in the N and O groups

We defined Ez as the effective modulus of trabeculae under a 1 N loading condition. The differentiation trends of the Ez and Ez ratios were highly similar to the mechanical properties of the 3DP VOI trabeculae, as described in the aforementioned compressive tests, as shown in Fig. 6. In addition, the reduction in Ez also demonstrated a consistent trend with the 3DP VOI trabeculae, as Table 3 shows. The FE model matched well with the mechanical compression results.

**Table 3:** Ez reduction of O group compare to N group

Anatomy	Properties	BV/TV reduction (%)	Ez reduction (%)
Medial condyles	Ez	16.07	44.19
Inter-condyles		25.06	66.02
Lateral condyles		15.75	47.12

### Linear regression

**Fig. 7:**
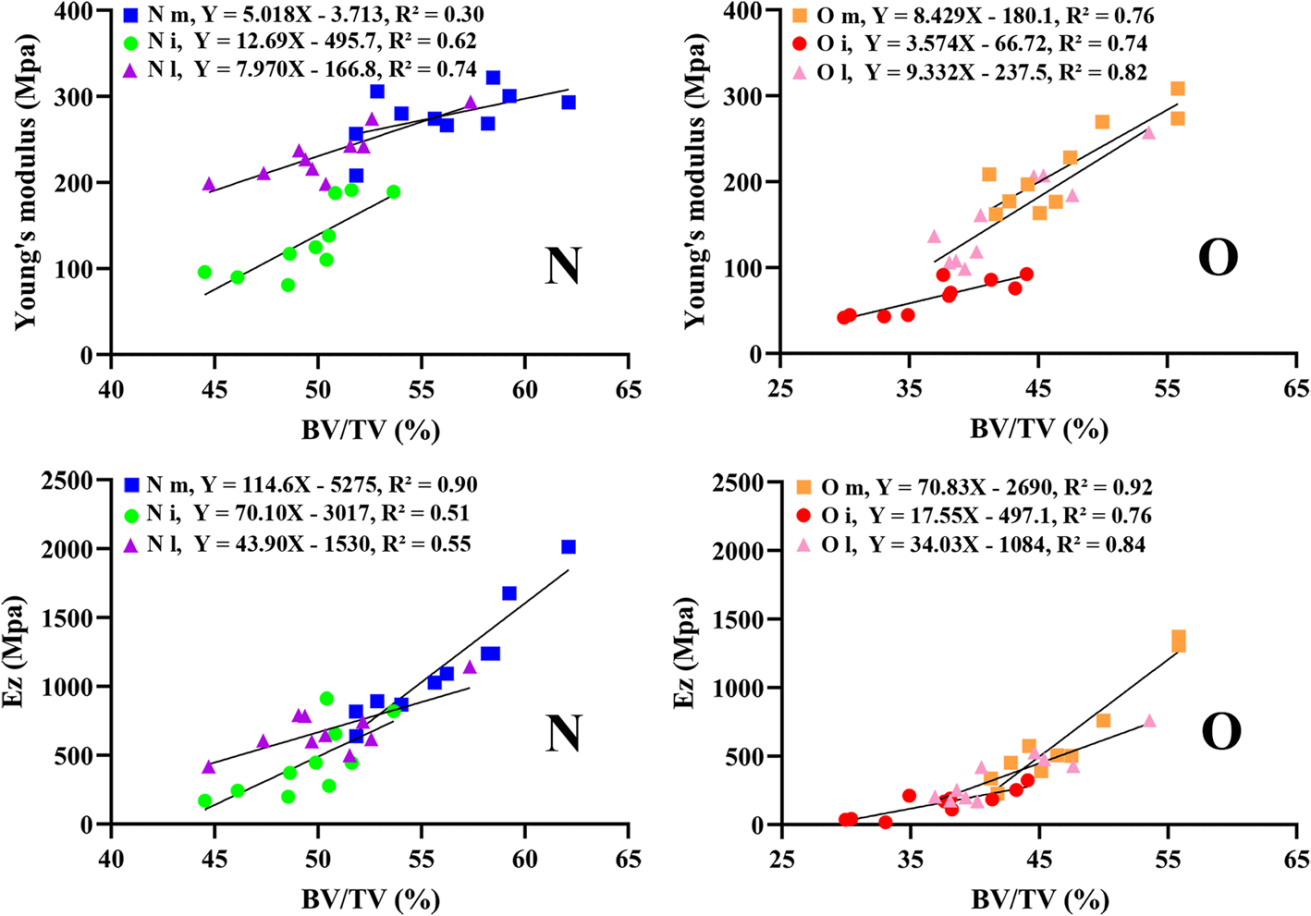
Linear regression between BV/TV and E, Ez in the N and O groups

BV/TV was found to be positively correlated with the Young's modulus and Ez. BV/TV was better at explaining the variance in the O group (R^2^ > 0.70) in both the compression test and FE simulation. The R^2^ value ranged from 0.30 to 0.90 in the normal group. In addition, FE results seems to be superior to match Ez (R^2^:0.51–0.92) compared to 3DP VOI trabeculae matching Young's modulus (R^2^:0.30–0.82), as shown in Fig. 7.

## Discussion

The structural parameters and mechanical properties of femoral condyle trabeculae were characterised in normal and ovariectomised rats to demonstrate the history of trabecular attenuation before and after ovariectomy. The femoral condyle was chosen for this study because it is more symmetrical and regular in anatomy than the proximal tibia. Therefore, it was ideal for controlling the potential anatomic interference to reveal the loading-induced trabecular alteration. This study quantified the spatial distribution and attenuation in femoral condyle trabeculae microarchitecture by combining 3DP and micro-CT-based FE model. The results demonstrated that the medial femoral condyle trabeculae dominated in BV/TV and the resultant mechanical properties in both healthy and ovariectomised conditions. The mechanical properties of trabeculae under ovariectomised conditions are more relevant to BV/TV. These changes indicate that the medial tibial plateau, corresponding to the medial femoral condyle, sustained more physiological load than the lateral plateau, regardless of ovariectomy.

In the O group, we found a significant decrease in BV/TV and Tb.N and a significant increase in Tb.Sp, whereas no significant deterioration was found in the DA in the three parts of the femoral condyle. These alterations indicated accelerated bone resorption, consistent with earlier studies on post-ovariectomy for 12-week-old rats [27, 28]. Specifically, the SMI (in general, perfect rod = 3 and perfect plate = 0) increased significantly only in the intercondyle (Fig. 1); a larger variation can be found in BV/TV, Tb.N, and Tb.Sp in the intercondyle compared to the medial and lateral condyles (Fig. 2); further, the weight and mechanical properties of the 3DP intercondyle trabeculae were also found to be minimal. These changes suggest that intercondyle trabeculae experience higher bone absorption compared to the lateral and medial condyles, confirming Wolff's law that in non-weight-bearing areas, trabeculae have less bone mass and a higher rate of bone resorption.

Previous studies investigating KOA involving the normal femoral condyle found that the medial condyle trabeculae BV/TV was slightly higher than that of the lateral condyle [29–31]. These anatomical differences were replicated in our study, and it was interesting to find these differences under osteoporosis conditions (represented by ovariectomy in the present study) (Fig. 2). In fact, previous studies, including the present study, demonstrated no statistically significant difference in BV/TV values between the medial and lateral condyle trabeculae; however, when referring to individual femurs, almost all femurs showed moderately higher BV/TV values in the medial condyle, indicating an inherent anatomical difference between the medial and lateral condyles. More than 70% of the variance in the Young's modulus of trabeculae can be explained by BV/TV [11, 32, 33], therefore, we must determine how far these tiny variances between the medial and lateral condyles contribute to the differentiation of mechanical properties.

The golden standard was to test the skeletal samples from cadavers or experimental animals; however, cadavers involve high cost and ethical issues and operation on experimental animals is challenging at the millimetre scale, limiting their applicability. 3DP [2, 34–37] and micro-CT based FE modelling [38–43] have been utilised to investigate the mechanical behaviour of trabeculae under different loading conditions and have shown an excellent prediction. Before testing 3DP VOI trabeculae, the key objective was to ensure that the 3D printer could accurately reproduce trabecular samples [36]. In the present study, the weight of 3DP VOI trabeculae from both groups significantly correlated with their BV/TV values (Table 1), indicating that the 3DP printer accurately printed the trabeculae models according to their BV/TV, and the 3DP VOI trabeculae were sufficiently accurate for the mechanical compression test.

Mechanical compression tests clearly indicated different trends in the Young's modulus, yield strength, ultimate strength, and stiffness, consistent with those of BV/TV (Fig. 3). Nevertheless, the medial and lateral condyles showed a relatively high standard deviation in these mechanical properties, which was reversed to the intercondyle, indicating a large variation among the individual subjects in these weight-bearing areas. As expected from the former BV/TV differentiation, the medial condyle had superior mechanical properties than the lateral condyle in healthy conditions (the Young's modulus, yield strength, ultimate strength, and stiffness were approximately 1.2, 1.3, 1.33, and 1.19 times that of the lateral condyle, respectively), and the superiority broadened under the osteoporosis condition (approximately 1.46, 1.38, 1.39, and 1.39 times, respectively) (Fig. 4). The FE simulation also demonstrates this gap: displacement and stress were found to more concentration on lateral condyle trabeculae (Fig. 5); the Ez of the medial condyle trabeculae was 1.68 and 1.78 times that of the lateral condyle before and after ovariectomy, respectively (Fig. 6). Previous studies identified the prevalence of medial compartment tibiofemoral joint loading during neutral landing patterns in hopping motion [44], as well as a higher bone mineral density for the healthy medial femoral condyle [45]. These experimental results support the findings of this study. Interestingly, a cadaver study identified a higher Young's modulus, yield strength, ultimate strength, and BMD in the lateral femoral condyle; however, their trabeculae samples were pooled along the anterior–posterior direction (compared to the vertical direction in the present study) [46], suggesting that the medial and lateral condyles sustain prevalent loading in different anatomical directions.

A decrease in BV/TV indeed leads to a drastic decline in mechanical properties, but depends on the anatomic region. As shown in Tables 2 and 3, the decreases in the BV/TV value in medial (16.07%) and lateral (15.75%) condyle trabeculae were very nearly the same; however, their corresponding decline in mechanical properties differed greatly, especially for the Young's modulus (22.02% vs. 32.42%), stiffness (21.76% vs. 30.37%), and Ez (44.19% vs. 47.12%). This decline was more critical in the intercondyle (with 25.06% lower BV/TV, 50.32% lower Young's modulus, and 66.02% lower Ez). Compared to the normal population, BV/TV decreases in osteoporotic individuals by 6.6% to 45% depending on the examined bone, such as vertebra, iliac bone, femoral head, distal femur, and distal radius [11]. The BV/TV varies significantly even in the same bone [27]. This range places our 15–25% decrease in BV/TV in the middle of the osteoporotic scale, possibly corresponding to the marked osteopenia stage. Similar to our results, another study identified that the BV/TV of femoral trabeculae ranged between 11 and 33% in osteoporotic individuals, corresponding to a decrease of approximately 20% in structural stiffness and 24% in structural strength [18]. Furthermore, a reduction of 8–10% in trabecular BV/TV results in an approximately 17% decrease in structural stiffness, according to an FE study [47]. These similar results support the validity of 3DP trabeculae and micro-CT-based FE model as dependable tools to predict the effect of trabecular attenuation on its mechanical properties.

Unexpectedly, the predicted accuracy of BV/TV in the variances of elastic properties was higher after ovariectomy (Fig. 7). This might be due to the loss of other microstructures that were not investigated in this study, such as trabecular connectivity density (Conn.D). Conn.D reflects small interconnecting trabeculae with small initial diameters, which were not reflected in the BV or BV/TV values. As bone volume decreases, there is a corresponding decrease in Conn.D, which may lead to a disproportionate loss of trabecular strength [48, 49], thereby augmenting the goodness of fit between BV/TV and elastic properties. However, because of the limited availability of young or premenopausal skeletal specimens [50], it remains to be demonstrated in the future.

This study had several limitations. How scale and organic material (i.e. polymer) affect the mechanical properties remains to be clarified. Furthermore, we looked at only one loading rate, which could be a potential limitation of our study. It is important to note that because we are studying the structural effects of trabecular architecture differentiation, the mechanical properties reported in our study are less informative (as the material tested is poly(lactic acid) (PLA) rather than bone tissue). However, the percentage change of the mechanical properties (and their similarity to previously reported findings) is informative.

## Conclusions

According to Wolff's law, loading triggers an adaptive bone remodelling process that, in the present study, may have higher trabecular BV/TV and better mechanical properties in the medial femoral condyle. The higher trabecular BV/TV and improved mechanical properties are due to a larger reaction force from the corresponding medial tibial plateau compared to the lateral plateau. This inherent imbalance appears to be aggravated after ovariectomy. Considering this, we can partly explain that varus knee deformities account for the majority of patients with KOA and affect more than 70% of patients with idiopathic KOA [19] and the "non-uniform settlement" phenomenon of the medial tibial plateau [25].

## Materials and methods

### Animal and samples

Twenty 8-week-old female Sprague–Dawley rats were randomly separated into two groups: normal control group (N, n = 10) and ovariectomised group (O, n = 10). Randomisation was performed using random numbers. They had no musculoskeletal disorders before the experiment. All rats were raised in a standard specific-pathogen-free environment and allowed free access to food. After 12 weeks, all rats (N, n = 10; O, n = 10) were euthanised by an overdose of anaesthesia, and the right femurs were dissected and collected. Femur samples were fixed with 4% paraformaldehyde for 48 h and kept at room temperature for subsequent micro-CT scans.

### Micro-CT tomography [27]

**Fig. 8:**
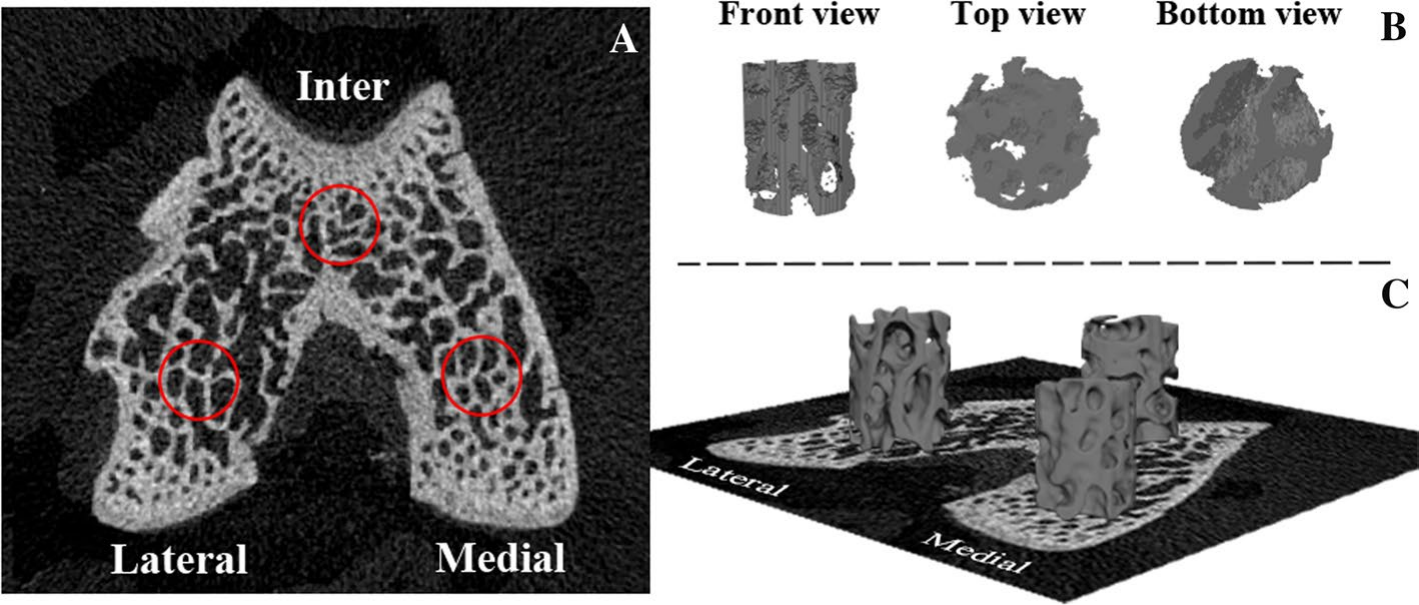
VOI in distal femoral condyle. **A** The bottom segmentation boundaries of VOI trabeculae. **B** Raw 3D model of VOI trabeculae containing spikes and unconnected elements. **C** Polished 3D model of VOI trabeculae

All excised femurs were imaged using a micro-CT scanner (SkyScan1172, Bruker, USA) at a resolution of 15 μm and with a voltage of 80 kV, current of 100 μA, an aluminium filter of 0.5 mm, rotation step of 0.6°, and exposure time of 360 ms. The images were then reconstructed using bundled Nrecon1.7.0.4. The DataViewer1.4.3 software (SkyScan1172, Bruker, USA) was employed to orient the cross-sectional images parallel to the transaxial plane. A cylindrical VOI with a diameter of 1 mm and height of 1.18 mm was placed in the weight-bearing regions of the medial and lateral femoral condyles. The intercondyle was also segmented for comparisons. The bottom segmentation boundaries were marked when the VOI diameter of the intercondyle was constrained by the endocortical bone margin enclosing the femoral trochlea and intercondylar fossa (Fig. 8A). CTAn1.16.8.0 (SkyScan1172, Bruker, USA) was used for the morphometric analysis of VOI trabeculae. A grey-scale threshold of 80–255 was chosen to segment the VOI as bitmap images. The measured 3D microstructural parameters were based on VOI trabeculae, including BV/TV, Tb.N, SMI, Tb.Th, Tb.Sp, and DA. The VOI trabeculae were also saved as a "stl" file for optimisation.

### 3D models optimisation

Since the raw the "stl" file format VOI trabeculae were full of noises (for example, spikes, unconnected elements) and were too rough for 3D printing and FE analysis (Fig. 8B), optimisation including smoothing, mesh reduction, and remeshing was applied in Geomagic Studio 2013(GEOMAGIC, USA). The optimisation was performed carefully to maintain structural integrity. 3D trabeculae were saved as "stl" files, and were ready for subsequent 3D printing (Fig. 8C).

### Three-dimensional printing

**Fig. 9:**
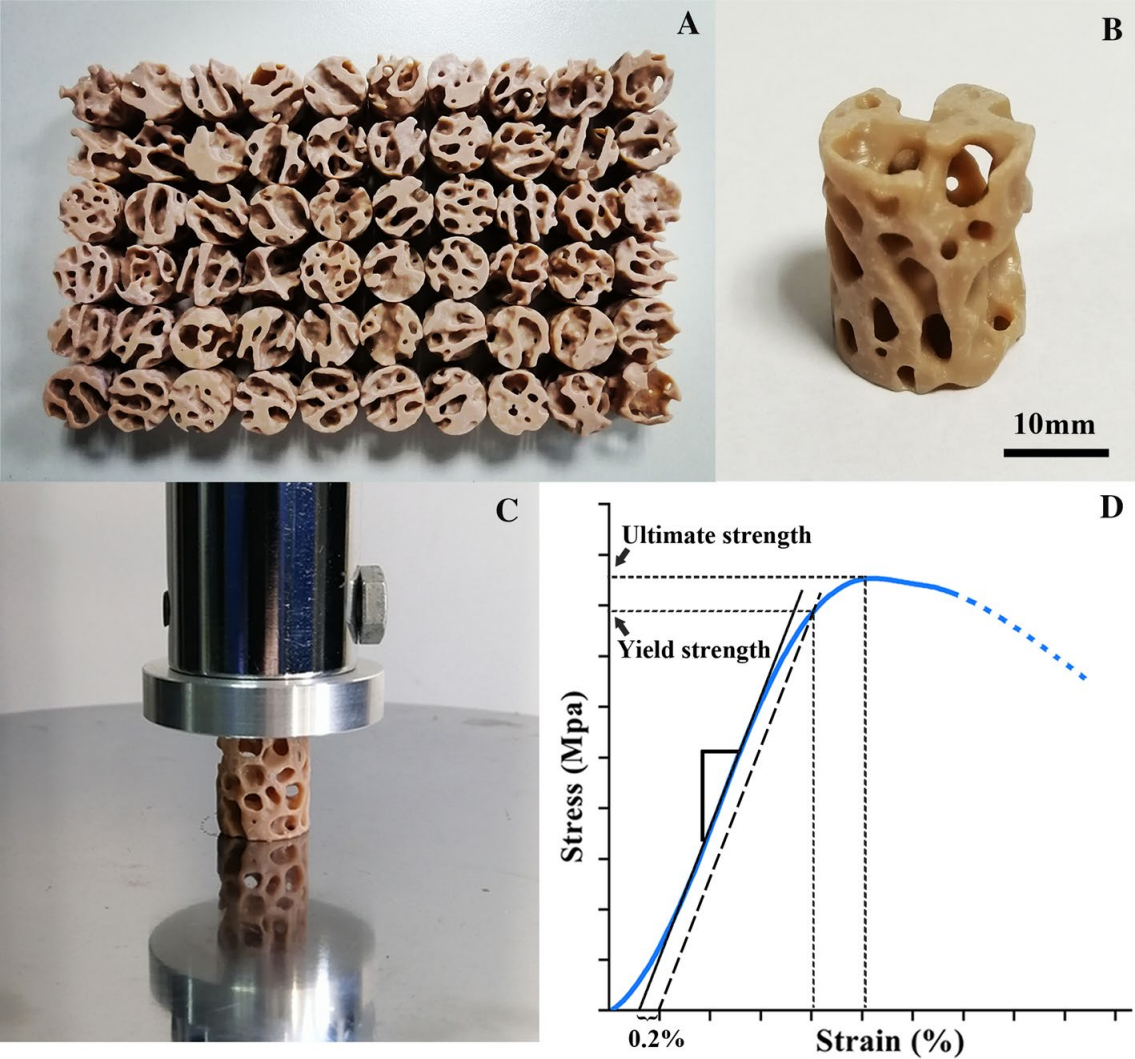
3DP VOI trabeculae and mechanical tests. **A** An overview of 3DP VOI trabeculae from femoral condyles. **B** A close-up view of 3DP VOI trabeculae. **C** A view of the universal testing machine used to load the 3DP VOI trabeculae in compression. **D** Typical stress–strain curve for the 3DP VOI trabeculae tested along the principal direction

3DP can accurately and precisely produce trabecular samples and produces informative mechanical alterations with simplicity and convenience [2, 35–37, 51]. The actual size of the VOI trabeculae was 1 mm (diameter) × 1.18 mm (height), which is too small to be fabricated using 3DP and to reproduce the microarchitecture of trabecular specimens. Therefore, the VOI trabeculae were scaled 20 times [52] using the Materialise Magics software (version 21.0; Materialise, Belgium). All VOI trabeculae were then printed using an HP Jet Fusion 3D printer (Hewlett-Packard, USA) using a PLA filament with a layer thickness of 60 μm (Fig. 9A, B), far smaller than the dimension of the magnified trabeculae. The HP jet fusion 3D printer uses a novel technique called multi-jet fusion. The technique provides high levels of part quality faster and cheaper than existing 3DP technologies [53, 54]. PLA has been extensively used to reproduce skeletal mechanical properties and has the advantages of biocompatibility and biodegradability [51, 55, 56]. According to the manufacturer, the melting temperature of the PLA filament is 170 – 230 °C, and the density is approximately 1.3 g/cm^3^. 3DP VOI trabeculae were weighed, and least squares linear regression was calculated against BV/TV to validate the accuracy of replicating microarchitecture [36].

### Mechanical tests

3DP VOI trabeculae (diameter 20 mm, height 23.6 mm) were loaded in uniaxial compression testing using a universal testing machine (Jingzhuo Machinery Factory, Yangzhou, China) equipped with a 10-kN load cell with an error of 0.3% of the indicated value and loaded at a rate of 2 mm/min. A small compression preload of 5 N was applied at the beginning of each experiment. The measurements were recorded every 100 ms (Fig. 9C). The Young's modulus, ultimate strength, yield strength, and stiffness were determined for the 3DP VOI trabeculae. Young's modulus was defined as the slope of the stress–strain curve in the linear region, while the ultimate strength was defined as the maximal stress. The yield strength was determined using the 0.2% offset method [57], and the stiffness was the slope of the load–deformation curve (Fig. 9D).

### FE modelling

The FE method was also used to predict the effective Young's modulus of VOI trabeculae. First, in Hypermesh 14.0 software (Altair, USA), post-optimised 3D trabeculae were meshed to C3D4 tetrahedral elements with a size of 18 μm. The average number of elements and nodes of the 3D trabeculae is shown in Table 4. The element density was kept sufficiently high to accurately represent the microstructure of the trabecular bone. The convergence test suggested that a size of 18 μm was sufficient for convergence.

**Table 4:** The average number of elements and nodes of 3D trabeculae (${\overline{\text{x}}} \pm {\text{s}}$)

Group	Anatomy	Element	Node
N	Medial condyle	321,635 ± 19,243	71,703 ± 3796
	Inter-condyle	275,052 ± 20,080	63,629 ± 3903
	Lateral condyle	283,878 ± 20,467	64,604 ± 4138
O	Medial condyle	274,169 ± 33,900	62,556 ± 6829
	Inter-condyle	218,647 ± 57,192	49,759 ± 7526
	Lateral condyle	253,848 ± 36,801	58,233 ± 7640

**Fig. 10:**
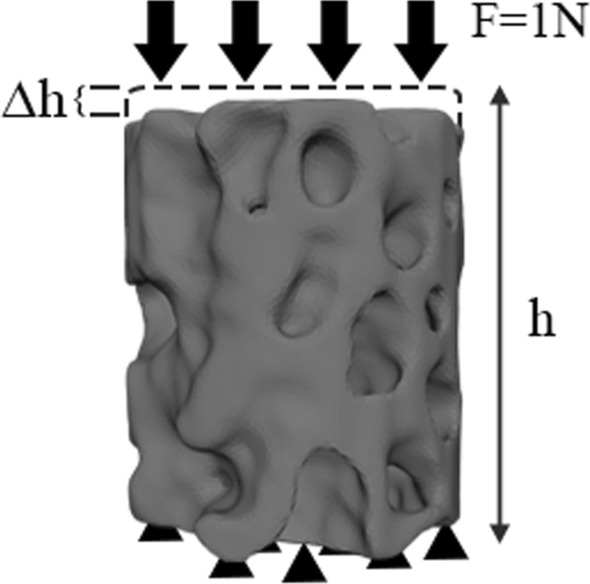
Finite element model of VOI trabeculae. Black arrows represent load using loading control; the black triangles represent boundary conditions

A linear elastic model was adopted, with a Poisson's ratio of 0.3. The Young's modulus of the elements was assigned according to the function derived from the distal femur of rats [33]:

$$ {\text{E }} = { 14899}\left( {{\text{BV}}/{\text{TV}}} \right)^{{{1}.{94}}} , $$

where E represents Young's modulus. A small uniaxial compressive force (F = 1 N, along the Z-axis) [58] was applied using loading control, and the resultant displacement was computed (Fig. 10). Let r, h, Δh, and F denote the radius, original height, maximal displacements, and applied force of the VOI trabeculae, respectively. The average stress can be calculated using F/πr^2^, and the average strain by Δh/h. Specifically, the cut-off values for the upper 95th percentile of the displacements along the Z-axis in each trabeculae were defined to represent the maximal displacements [59, 60]. The maximum displacement was calculated by averaging nodal displacements. Therefore, the effective Young's modulus [9] of VOI trabeculae can be calculated using the following equation:

$$\mathrm{E}=\frac{\frac{\mathrm{F}}{\uppi {\mathrm{r}}^{2}}}{\frac{\mathrm{\Delta h}}{\mathrm{h}}}.$$

Using this approach, the effective Young's modulus of the VOI trabeculae along the Z direction, denoted as Ez, was obtained.

### Statistical analysis

We must point out that because we use PLA rather than bone tissue, the mechanical properties may differ from the actual bone material. However, the sample ratio still demonstrates the difference and attenuation of their mechanical behaviour. The structural parameters and mechanical properties of the N and O groups are compared. We calculated the ratios of the structural parameters and mechanical properties within the samples. The Mann–Whitney U test (SPSS20.0, IBM, USA) was used to identify significant differences. Linear regression was also performed to describe the correlation between the structural parameters and mechanical properties.

## Data Availability

The datasets used and/or analysed during the current study are available from the corresponding author on reasonable request.
